# Beyond Positive Affect: Discrete Positive Emotions Differentiate Major Depression from Social Anxiety Disorder

**DOI:** 10.1007/s10608-023-10355-y

**Published:** 2023-02-13

**Authors:** Angela A. Chin, Alison M. Sweet, Charles T. Taylor

**Affiliations:** Department of Psychiatry, University of California San Diego, La Jolla, CA, 92037 San Diego, USA

**Keywords:** Social anxiety, Depression, Positive emotion, Affect, Discrete emotions

## Abstract

**Background:**

Social anxiety disorder (SAD) and major depressive disorder (MDD) are both associated with diminished global positive affect. However, little is known about which specific positive emotions are affected, and which positive emotions differentiate MDD from SAD.

**Methods:**

Four groups of adults recruited from the community were examined (*N* = 272): control group (no psychiatric history; *n* = 76), SAD without MDD group (*n* = 76), MDD without SAD group (*n* = 46), and comorbid group (diagnoses of both SAD and MDD; *n* = 74). Discrete positive emotions were measured with the Modified Differential Emotions Scale, which asked about the frequency of 10 different positive emotions experienced during the past week.

**Results:**

The control group had higher scores on all positive emotions compared to all three clinical groups. The SAD group had higher scores on awe, inspiration, interest, and joy compared to the MDD group, and higher scores on those emotions, as well as amusement, hope, love, pride, and contentment, than the comorbid group. MDD and comorbid groups did not differ on any positive emotions. Gratitude did not differ significantly between clinical groups.

**Conclusion:**

Adopting a discrete positive emotion approach revealed shared and distinct features across SAD, MDD, and their comorbidity. We consider possible mechanisms underlying transdiagnostic vs. disorder-specific emotion deficits.

**Supplementary Information:**

The online version contains supplementary material available at 10.1007/s10608-023-10355-y.

Social anxiety disorder (SAD) and major depressive disorder (MDD) are common mental health conditions that lead to impairment in social and occupational functioning (5th ed.; DSM-5; American Psychiatric Association, 2013). These conditions frequently co-occur, and their co-occurrence is associated with stronger symptom severity and impairment, and poorer prognosis and treatment response compared to these disorders in isolation (Adams et al., 2016; Ballenger, 2000; Gorman, 1997). Thus, it is crucial to understand both shared and distinct features of these conditions. One such feature that is integral to both SAD and MDD is affect. It was initially hypothesized that increased negative affect was shared across anxiety and depressive disorders, whereas diminished positive affect was unique to depression (Clark & Watson, 1991). However, evidence consistently demonstrates that SAD is characterized by positive affect deficits that are comparable in magnitude to those observed in MDD (Brown et al., 1998) and are not accounted for by co-occurring symptoms of depression (Kashdan, 2007). Despite the growing literature on diminished positive affect in emotional disorders, there is a need for more research to identify which specific positive emotions are associated with these disorders (e.g. joy, gratitude, awe, contentment, pride, love; Vazquez 2017). Studying discrete positive emotions may uncover shared and distinct emotion profiles across SAD and MDD that have been obscured by the prevailing global positive affect perspective – observations that could advance descriptive phenomenology of each condition and the approach to assessment and treatment.

Fredrickson’s (1998) broaden-and-build theory provides an empirically supported framework for understanding the functional importance of positive emotions in supporting psychological health and well-being (Fredrickson, 2013). Shiota et al., (2017) expanded on this framework, summarizing evidence that different positive emotions are associated with partially unique patterns of cognition, physiological reactivity, and behavior that facilitate the acquisition of social, intellectual, or material resources. For example, love signifies the opportunity for affiliation and intimacy (Shiota et al., 2017); it motivates a person to approach others and establish close relationships. Pride, which arises after a personal achievement, acts as an indicator of high social status (Tracy et al., 2013). It motivates individuals to share their achievements with others, and to seek out intellectual and social resources to accomplish their goals. It may be valuable, therefore, to identify which specific positive emotions are diminished in SAD and MDD so that treatments can target those emotions and their associated behaviors.

A few studies have examined which positive emotion deficits may be related to depressive symptoms. Gruber and colleagues (2011) found that in college students, current depressive symptoms were most strongly predicted by lower levels of trait pride as opposed to happiness or amusement. The second study had students watch pride-, happy-, and amusement-eliciting films and rate how much pride, happiness, and amusement they experienced during each clip. The depressive symptom group reported significantly less happiness than the non-depressive symptom group after watching the pride film. There were no other significant differences, reinforcing the unique association between pride and depressive symptoms. In another study examining adolescents diagnosed with bipolar disorder, Gruber and colleagues (2017) found that lower levels of joy were more strongly associated with depressive symptoms compared to interest and surprise. Together these papers suggest that deficits in pride and joy may be particularly associated with depressive symptoms.

Other studies have examined how social anxiety may be associated with certain discrete positive emotions. Cohen & Huppert (2018) found that pride, joy, love, awe, amusement, and contentment significantly correlated with social anxiety symptoms, whereas compassion did not. Out of these positive emotions, only deficits in pride and love uniquely predicted social anxiety beyond variance accounted for by the remaining positive emotions and symptoms of depression. When including global positive affect in the regression models with pride and joy – and after controlling for depression – only lower levels of pride predicted social anxiety symptoms, whereas global positive affect and joy did not. In their second study, the experience of pride better predicted social anxiety than the expression of pride. The final study revealed that individuals diagnosed with SAD experienced lower levels of pride than low-anxiety control subjects, and pride uniquely predicted SAD diagnostic status beyond variance accounted for by global positive affect and depression. Together, those findings converge with other work demonstrating that social anxiety is inversely associated with pride in clinical samples other than SAD (i.e., MDD; Gilbert 2000) and across a broad range of samples measuring pride and social anxiety (for a recent meta-analysis, see Dickens & Robins 2020).

To summarize, prior studies suggest that deficits in pride are most consistently characteristic of both SAD and MDD, and diminished joy and love may also be associated with these conditions. These studies underscore the potential value of evaluating discrete positive emotions and hint at shared and unique emotion experiences across disorders. However, they assessed a limited number of discrete positive emotions and did not directly compare emotion experiences in SAD versus MDD. The current study thus sought to replicate and extend previous findings by measuring a broader array of positive emotions, by comparing SAD to MDD, and by studying a large, more demographically diverse community sample. The primary research questions were: (1) Do individuals with SAD, MDD, or both SAD and MDD experience specific positive emotions less often than individuals without a history of an anxiety or depression diagnosis? and (2) Does the frequency of positive emotion experiences differ across individuals with SAD, MDD, or comorbid SAD and MDD? Based on Cohen & Huppert (2018) and Gruber et al., (2011), we hypothesized that people with a diagnosis of SAD or MDD would experience less pride than the control group; people with a diagnosis of SAD would experience less love than the control group; and those with a diagnosis of MDD would experience less joy than the control group. Given limited research on other positive emotions and comparisons between SAD, MDD, and their comorbidity, we did not have any further predictions. Also, because culture can lead to differences in positive emotion experience (Ishii & Eisen, 2021; Polanco-Roman et al., 2019; Senft et al., 2021), we explored whether participant cultural heritage influenced the hypothesized group differences in positive emotions.

## Methods

### Participants

The sample included 272 participants recruited from the community for four previously conducted clinical trials and one cross-sectional study. These trials selected for individuals with either (1) a current principal diagnosis of SAD (NCT02136212) or (2) a current principal diagnosis of MDD (confirmed with the Mini International Neuropsychiatric Interview [MINI; Sheehan et al., 1998])^1^ (NCT02330744), (3) clinically elevated anxiety symptoms (score ≥ 8 on the Overall Anxiety Severity and Impairment Scale [OASIS; Norman et al., 2006]) and/or elevated depressive symptoms (score ≥ 10 on the Patient Health Questionnaire-9 [PHQ-9; Kroenke & Spitzer, 2001]; NCT03196544; NCT02330627; the latter study also required evidence of social disconnection and impairment, i.e., score < 90 on the Social Connectedness Scale – Revised [SCSR; Lee et al., 2001] and score ≥ 5 on the social domain of the Sheehan Disability Scale [SDS; Leon et al., 1997]), or (4) no current or past psychiatric diagnoses assessed using the MINI and a score < 20 on the Liebowitz Social Anxiety Scale (LSAS; Liebowitz 1987). All participants had to be between ages 18 to 55 and have sufficient English skills to understand the procedures and provide informed consent.

Participants who met any of the following criteria were excluded to avoid confounding the results of the parent studies and to ensure participants’ safety when completing the study procedures: (1) current pharmacological treatment for anxiety or depression (e.g. antidepressants, benzodiazepines), (2) concurrent psychotherapy unless 12 week stability criteria had been met, (3) current active suicidal ideation with intent, (4) history of a major neurological disorder or moderate to severe traumatic brain injury, (5) moderate or severe alcohol or marijuana use within the past year, or mild or greater substance use disorder of all other drugs within the past year, (6) bipolar I or psychotic disorders, and (7) characteristics that may compromise MRI safety (e.g. metal in the body).

The diagnostic makeup of this sample consisted of 27.9% individuals with a SAD principal diagnosis and no MDD diagnosis (*n =* 76); 16.9% of individuals with a MDD principal diagnosis and no SAD diagnosis (*n =* 46); 27.2% individuals with SAD and MDD comorbid diagnoses (*n =* 74); and 27.9% individuals in the control group (*n =* 76). The demographics of this sample were: age (*M* = 25.68, *SD* = 7.75), years of education (*M* = 14.39, *SD* = 3.42), gender (37.5% male, 60.7% female, 1.5% neither), race (30.9% Asian, 4.8% Black, 2.2% Hawaiian Native/Pacific Islander, 0.4% Native American/Alaskan Native, 46.3% White, 8.8% more than one race, 3.7% other, and 2.2% unknown or declined to respond), ethnicity (22.1% Hispanic), and race and ethnicity combined for cultural heritage (35.9% Asian heritage, 39.3% European heritage, 24.8% Latin heritage; Senft et al., 2021).^2^ See Table 1 for demographic breakdown by group.

**Table 1:** *LSAS and BDI-II Scores and Demographics by Group*

Symptom Measure		Diagnosis							
		Control (*n* = 76)		SAD only (*n* = 76)		MDD only (*n* = 46)		Comorbid (*n* = 74)	
		*M*	*SD*	*M*	*SD*	*M*	*SD*	*M*	*SD*
LSAS^a^		9.49	6.33	76.32	16.69	49.52	23.68	81.05	22.69
BDI-II^b^		1.73	2.27	15.37	9.20	27.88	8.25	30.33	9.52
Demographic		*M*	*SD*	*M*	*SD*	*M*	*SD*	*M*	*SD*
Age		25.31	8.16	23.78	5.86	29.48	10.54	25.64	6.15
Years of education		15.71	2.73	14.64	2.77	13.67	4.05	13.23	3.76
Gender		*n*	%	*n*	%	*n*	%	*n*	%
Men		24	31.6	31	40.8	18	39.1	29	39.2
Women		51	67.1	45	59.2	27	58.7	42	56.8
Neither		0	0.0	0	0.0	1	2.2	3	4.1
Ethnicity									
Not Hispanic		62	81.6	61	80.3	34	73.9	53	71.6
Hispanic		13	17.1	15	17.1	11	23.9	21	28.4
Race									
Asian		32	42.1	29	38.2	7	15.2	16	21.6
Black		3	3.9	3	3.9	2	4.3	5	6.8
Hawaiian Native or Pacific Islander		1	1.3	4	5.3	0	0.0	1	1.4
Native American or Alaska Native		0	0.0	1	1.3	0	0.0	0	0.0
White		30	39.5	34	44.7	25	54.3	37	50.0
More than one race		6	7.9	3	3.9	5	10.9	10	13.5
Other		2	2.6	1	1.3	4	8.7	3	4.1
Unknown or decline to respond		1	1.3	1	1.3	2	4.3	2	2.7
Cultural heritage: Race and ethnicity combined									
Asian heritage		32	42.1	29	38.2	7	15.2	16	21.6
European heritage		22	28.9	24	31.2	20	43.5	26	35.1
Latin heritage		13	17.1	15	19.7	10	21.7	20	27.0

*Note. N =* 272. Some participants had missing data. The number of participants with LSAS and BDI-II scores were: LSAS (*n* = 270), and BDI-II (*n* = 225). The number of participants with demographic data were as follows: age (*n* = 271), years of education (*n* = 270), gender (*n* = 271), ethnicity (*n* = 270), race (*n* = 270), and race and ethnicity combined (*n* = 234)

^a^ The Liebowitz Social Anxiety Scale (LSAS; Liebowitz 1987) ranges from 0 to 144

^b^ The Beck Depression Inventory (BDI-II; Beck et al., 1996) ranges from 0 to 63

### Measures

#### Discrete Positive Emotions

To measure positive emotions, participants completed the Modified Differential Emotions Scale (mDES; Fredrickson et al., 2003). The mDES has 20 items measuring 10 discrete negative emotions (e.g. guilt, stress) and 10 discrete positive emotions (i.e., amusement, awe, gratitude, hope, inspiration, interest, joy, love, pride, contentment). Participants rated how often they experienced each emotion within the past week on a scale of 0 to 4 (0 being “Never” and 4 being “Most of the time”). For the purposes of this study, only positive emotions were included in analysis.

#### Social Anxiety Symptoms

The Liebowitz Social Anxiety Scale (LSAS; Liebowitz 1987) was used to measure the severity of social anxiety symptoms. Both the self-report and clinician-administered versions were used in these clinical trials, and the self-report version has been shown to be comparable to the clinician-administered version (Fresco et al., 2001). Participants rated their level of fear and avoidance for 24 social situations on a 4-point scale ranging from “none/never” to “severe/usually.” The total score indicates severity of social anxiety.

#### Depression Symptoms

The Beck Depression Inventory II (BDI-II; Beck et al., 1996) was used to measure the severity of depressive symptoms. The BDI-II has 21 multiple-choice items that assess symptoms of depression, such as loss of pleasure and self-dislike, from the past two weeks. Individuals rated each item from 0 to 3, and the total score indicates severity of depression. This measure has been shown to be a reliable and well-validated measure of depressive symptoms (Beck et al., 1996; Dozois et al., 1998).

### Procedure

Participants were recruited through public flyers and online advertisements, and from mental health outpatient clinics and primary care centers. After expressing interest, they were contacted via phone to learn information regarding the study and determine their initial eligibility. Participants then attended a final eligibility assessment, which included providing written informed consent and completing a diagnostic interview and symptom surveys. Eligible participants were then given a battery of self-report measures to complete, including the mDES.

### Statistical Analyses

#### Preliminary Analyses

Demographics, LSAS scores, and BDI-II scores were assessed for possible differences between groups.

#### Primary Analysis

A multivariate analysis of variance (MANOVA) was conducted to examine group differences in positive emotions. The dependent variables were the 10 positive emotions from the mDES. Univariate tests were examined for each discrete positive emotion pending a significant omnibus diagnostic group effect. Tukey’s HSD was used to compare positive emotions between groups as follows: SAD principal diagnosis, no MDD (*n =* 76); MDD principal diagnosis, no SAD (*n =* 46); SAD and MDD comorbid (*n =* 74); and the control group (*n =* 76).

#### Secondary Analyses

Given that diagnoses can place artificial boundaries on symptoms that exist dimensionally, a secondary analysis was conducted examining zero-order correlations between LSAS scores, BDI-II scores, and discrete positive emotions, as well as partial correlations for each symptom measure accounting for variance in the other symptom domain. 3

Race and ethnicity were combined as a fixed factor (Latin heritage vs. European heritage vs. Asian heritage; Senft et al., 2021) in a separate subsequent MANOVA to explore whether hypothesized diagnostic group differences in positive emotions varied by cultural heritage.

## Results

### Preliminary Analyses

Gender (*X*^*2*^(6) = 7.523, *p* = .275), ethnicity (*X*^*2*^(3) = 3.060, *p* = .382), and race (*X*^*2*^(21) = 30.325, *p* = .086) did not differ significantly across groups. Age differed such that the MDD only group was older than the control, SAD only, and comorbid groups (*F*(3, 267) = 5.537, *p* = .001). Years of education also differed such that the control group had more years of education than the MDD only and comorbid groups, and the SAD only group had more years of education than the comorbid group (*F*(3, 266) = 7.941, *p* < .001). See Table 1. The main analysis was repeated with age and years of education as covariates. The pattern of findings did not change when age and years of education were entered as covariates. Therefore, only the models without covariates are reported in the main text.

LSAS scores differed between the groups (*F*(3, 266) = 245.026, *p* < .001) such that all clinical groups had higher scores than the control group, and the comorbid and SAD only groups had greater scores than the MDD only group; scores did not differ significantly between the SAD only and comorbid groups. BDI-II scores also differed between groups (*F*(3, 221) = 176.653, *p* < .001). Comorbid and MDD only groups had higher BDI-II scores than the SAD only and control groups, and the SAD group had a greater score than the control group. The comorbid and MDD only groups’ scores did not differ significantly. See Table 1 for LSAS and BDI-II mean scores by group.

### Primary Analysis

The main analysis revealed that positive emotions differed across SAD only, MDD only, comorbid, and control groups (*F*(30, 760.892) = 13.566, *p* < .001; Wilk’s Λ = 0.284; η_p_^2^ = 0.342). See Table 2 for descriptive summaries. Follow-up Tukey’s HSD tests indicated that the control group experienced all positive emotions more than the clinical groups (SAD only, MDD only, and comorbid; all *p* < .05). The comorbid group experienced amusement, awe, hope, inspiration, interest, joy, love, pride, and contentment less than the SAD only group, and the MDD only group experienced awe, inspiration, interest, and joy less than the SAD only group (all *p* < .05). The MDD only and comorbid groups did not differ across any positive emotion (all *p* > .05). Gratitude was the only positive emotion not to significantly differ between clinical groups. See Table 2 for descriptive summaries and Supplemental Table 1 for between-group effect sizes.

**Table 2:** *Mean Discrete Positive Emotion Scores by Group*

	Control		SAD only		MDD only		Comorbid		
Discrete Positive Emotions	M	SD	M	SD	M	SD	M	SD	η_p_^2^
Amusement	2.00_a_	0.88	1.13_b_	0.87	0.76_bc_	0.77	0.69_c_	0.76	0.30
Awe	1.55_a_	0.93	0.67_b_	0.79	0.30_c_	0.55	0.35_c_	0.58	0.32
Gratitude	2.36_a_	0.76	1.50_b_	0.95	1.26_b_	0.80	1.14_b_	1.01	0.23
Hope	2.26_a_	0.75	0.88_b_	0.83	0.54_bc_	0.69	0.42_c_	0.64	0.52
Inspiration	1.96_a_	0.86	0.70_b_	0.78	0.24_c_	0.52	0.32_c_	0.58	0.49
Interest	2.26_a_	0.77	1.12_b_	0.91	0.52_c_	0.62	0.62_c_	0.75	0.44
Joy	2.41_a_	0.79	1.05_b_	0.88	0.48_c_	0.59	0.35_c_	0.53	0.57
Love	2.58_a_	0.59	1.05_b_	0.91	0.89_bc_	0.88	0.65_c_	0.87	0.49
Pride	2.16_a_	0.78	0.64_b_	0.81	0.46_bc_	0.62	0.23_c_	0.48	0.56
Contentment	2.09_a_	0.84	0.75_b_	0.80	0.41_bc_	0.50	0.32_c_	0.58	0.52

*Note.* Means sharing the same subscript are not significantly different from each other (Tukey’s HSD, *p* < .05)

### Secondary Analyses

#### Dimensional Analyses

All positive emotions had significant negative correlations with LSAS and BDI-II scores (all *p* < .001). When controlling for depression, amusement, gratitude, and interest no longer correlated with social anxiety (all *p* > .05). When controlling for social anxiety, all positive emotions still correlated with depression (all *p* < .001). See Supplemental Table 2 for correlations. See Supplemental Fig. 1 and Supplemental Fig. 2 for frequency distributions of LSAS and BDI-II scores.

#### Cultural Heritage Analyses

Positive emotions did not differ across combined race and ethnicity (*F*(20, 426) = 1.391, *p* = .122; Wilk’s Λ = 0.881; η_p_^2^ = 0.061). Race and ethnicity also did not interact significantly with diagnostic groups, meaning cultural heritage did not impact the effect of diagnostic group on positive emotions (*F*(60, 1121.029) = 0.849, *p* = .787; Wilk’s Λ = 0.792; η_p_^2^ = 0.038). These results should be interpreted cautiously because cultural heritage groups were created post hoc and differed significantly across diagnostic groups (*X*^*2*^(6) = 13.826, *p* = .032). See Table 1.

## Discussion

The current study examined potential differences in discrete positive emotions between individuals meeting diagnostic criteria for SAD, MDD or those without. The SAD, MDD, and comorbid groups displayed deficits relative to controls across all discrete positive emotions – consistent with prior literature pointing to global diminished positive affect in these conditions (Brown et al., 1998; Kashdan, 2007). Previous studies suggested that pride in particular may be robustly diminished in SAD and MDD, with reduced experiences of love (in SAD) and joy (in MDD) also characterizing these conditions compared to other positive emotions (Cohen & Huppert, 2018; Gruber et al., 2011). In the current sample, these were the emotions that displayed larger effect sizes relative to most other positive emotions, further buttressing their prominence in these conditions. This study also contributes novel findings regarding amusement, awe, gratitude, hope, inspiration, interest, and contentment, which were experienced less in those diagnosed with SAD or MDD compared to the control group. Direct comparisons between diagnostic groups revealed that awe, inspiration, interest, and joy were experienced less in those diagnosed with MDD (with or without SAD) compared to SAD only, whereas love, pride, contentment, and amusement did not differ between MDD only and SAD only groups but were significantly lower in the comorbid group compared to the SAD only group. These differences in positive emotion experiences between clinical groups highlights the potential value of moving beyond global positive affect assessments, and implies that depression may be characterized by broad positive emotion deficits whereas the most pronounced deficits in social anxiety may lie within specific emotion domains that are further exacerbated by depression comorbidity.

The discrete positive emotions experienced less in both SAD and MDD only groups and to a similar degree (i.e., gratitude, love, and pride) primarily serve social functions. Gratitude is a response to unexpected benefits due to another person’s actions, and it motivates individuals to express kindness and generosity to others (McCullough et al., 2001; Shiota et al., 2017). Love is a response to interpersonal connection, affiliation, and intimacy. It motivates individuals to build social bonds (Fredrickson, 2013; Shiota et al., 2017). Pride is a response to the opportunity of high social status and accomplishing socially valued outcomes. It creates motivation in individuals to aspire for more achievements and to increase their social rank (Williams & DeSteno, 2008). Deficits in these specific positive emotions may be the result of disorder specific symptoms within social contexts (e.g., social avoidance due to fears of negative evaluation in SAD or avolition in MDD). They may also contribute to impairments in social relationship functioning that are common across both conditions. Research is needed to examine those possibilities.

Amusement, contentment, and hope also did not differ significantly between SAD and MDD only groups; however, effect size differences were moderate (0.45 to 0.51; SAD > MDD). Amusement is associated with opportunities for humor and play (Shiota et al., 2017), and it motivates individuals to share laughter and strengthen social bonds (Gervais & Wilson, 2005). Contentment is a response to when one’s circumstances are perceived as satisfactory (Fredrickson, 2013), and hope serves to build optimism and resilience in response to grim circumstances – motivating people to change their situation (Lazarus, 1991). Deficits in these emotions may reflect limited expression of positive emotions (amusement), general life dissatisfaction (contentment), and ruminative tendencies that hinder positive, future-oriented cognition (hope) associated with MDD and SAD (Dryman & Heimberg, 2018; Eng et al., 2005; Judd et al., 2000; Starr & Davila, 2012).

Inspiration, interest, joy, and awe were experienced less in the MDD and comorbid groups compared to the SAD only group (effect size differences were medium to large). Inspiration is an emotion that pushes people to challenge themselves and to grow (Algoe & Haidt, 2009; Thrash & Elliot, 2004). Interest similarly prompts people to learn, explore, and expand their knowledge within novel circumstances (Izard, 1977; Silvia, 2008). Joy is associated with play, experiential learning, and unexpected good fortune (Fredrickson, 2013). Awe is an emotion tied to expanding one’s worldview to accommodate vast, information-rich stimuli (Griskevicius et al., 2010; Keltner & Haidt, 2003). These emotions may relate in part to anhedonia, which is a defining characteristic of MDD. Anhedonia is the inability to experience pleasure, and it leads to less motivation to pursue goals and seek out novel or enjoyable experiences. Inspiration and interest are goal- or future-oriented, and joy is related to pleasure – features that may explain why the experience of these emotions is particularly reduced in individuals with MDD compared to SAD alone. This pattern may also suggest that high arousal positive emotions are diminished to a greater extent in MDD than in SAD.

The secondary dimensional analyses largely converged with the primary results. All positive emotions significantly correlated with both depression and social anxiety. When accounting for shared variance in symptoms, all positive emotions remained correlated with depression after controlling for social anxiety; in contrast, amusement, gratitude, and interest no longer significantly correlated with social anxiety after controlling for depression. These findings further suggest that overall deficits in positive emotions may be associated with depression, whereas social anxiety appears to be characterized by specific positive emotion deficits. Further research is needed on the association between discrete positive emotions and continuous measures of social anxiety and depression, as participants in this study were recruited for distinct diagnostic groups as opposed to dimensional symptomology.

Overall, the findings are largely consistent with previous studies (Cohen & Huppert, 2018; Gruber et al., 2011, 2017) – demonstrating that pride and love are reliably diminished in both SAD and MDD, whereas joy appears most prominently diminished in MDD. Also consistent with prior work were associations between awe and contentment with both social anxiety and depression (Cohen & Huppert, 2018), and amusement with social anxiety (Cohen & Huppert, 2018) and depression (Gruber et al., 2011). Amusement did not, however, correlate with depression in Cohen & Huppert (2018), and interest did not correlate with depression in Gruber et al., (2017). Inconsistent positive emotion findings across studies may be attributed to differences in samples (e.g., clinical vs. student; adults vs. adolescents) or scales used to measure discrete positive emotions. Nevertheless, the consistency of some positive emotion findings across studies (e.g., pride, love, joy) further underscores their potential importance in understanding the pathophysiology of these conditions.

The exploratory analyses indicated that culture did not interact with diagnosis nor contribute to differences in positive emotions. Further investigation is needed, since participants were not recruited with these characteristics in mind. Race and ethnicity alone do not wholly reflect culture, and Hispanic and Latin heritage are not synonymous with each other. There are also other cultural aspects that may interact with diagnosis and positive emotions, such as degree of acculturation and identification with one’s native culture, or the desirability and appropriateness of certain emotions in different communities (Koneru et al., 2007; Polanco-Roman et al., 2019; Senft et al., 2021). Additionally, culture and language are embedded in the construction of emotions, which can lead to different emotional experiences (Ishii & Eisen, 2021). The current study is limited by the mDES, since it was developed and validated within English-speaking, American samples. Thus, future studies should recruit larger and more representative samples to study how aspects of race, ethnicity, and culture may influence the experience of positive emotions in affective disorders, and consider different methods of measuring emotion.

Beyond cultural considerations, using the mDES to measure emotions has other limitations. It utilizes single-item, self-report ratings, and therefore requires individuals to identify and communicate their emotions. Since there are ways to process emotion other than verbally (e.g., somatically; Villanueva et al., 2021), and single-item ratings can limit how much information is gathered, future studies should measure emotions in ways better able to capture their complexity. Behavioral tasks (i.e., emotion challenges) may also yield more ecologically valid findings than self-reports. Another limitation is that participants were recruited separately for different parent studies – yielding uneven sample sizes across groups. Despite these limitations, the current findings suggest that moving beyond a global positive affect perspective has the potential to advance a more precise understanding of positive emotion deficits in social anxiety and depression – findings that could inform greater treatment specificity and personalization.

## Electronic Supplementary Material

Below is the link to the electronic supplementary material.

Supplementary Material 1

Supplementary Material 2

Supplementary Material 3

Supplementary Material 4

## Data Availability

The data that supports the findings of this study are available from the corresponding author upon reasonable request.

## Footnotes

1 Version 5.0.0 was used for some participants, and version 7.0.0 was used for later participants when it became available. Version 5.0.0 is based on DSM-IV diagnostic definitions, and version 7.0.0 is based on the DSM-5. Interviews using version 5.0.0 were later scored to reflect DSM-5 criteria.

2 Asian heritage (*n* = 84) was defined as participants who identified as Asian. European heritage (*n* = 92) was defined as participants who identified as White and not Hispanic. Latin heritage (*n* = 58) was defined as participants who identified as Hispanic and not White, or White and Hispanic. Participants who did not identify with one of those categories were not included in the exploratory cultural heritage analysis.

3 We thank the anonymous reviewer for suggesting these analyses.
