# Supplementary material for: Beyond Positive Affect: Discrete Positive Emotions Differentiate Major Depression from Social Anxiety Disorder
Source: Cognit Ther Res. 2023 Feb 13;47(3):377–85. doi: 10.1007/s10608-023-10355-y (PMC10164670; doi:10.1007/s10608-023-10355-y)
Supplement: Supplementary file 3 — Supplementary Material 3 [file 10608_2023_10355_MOESM3_ESM.pdf]

Springer
the language of science

Conflict-of-Interest Disclosure Form

Cognitive Therapy and Research

When an author or the institution of the author has a relationship, financial or otherwise, with individuals or organizations that could influence the author's work inappropriately, a conflict of interest may exist. Examples of potential conflicts of interest may include but are not limited to academic, personal, or political relationships; employment; consultancies or honoraria; and financial connections, such as stock ownership and funding. Although an author may not feel that there are conflicts, disclosure of relationships and interests that could be viewed by others as conflicts of interest affords a more transparent and prudent process.

All authors and co-authors (if any) of papers submitted to *Cognitive Therapy and Research* must complete this form and disclose any actual or potential conflict of interest. The journal may publish such disclosures. Please complete and return this form (one per author) and submit it/them **together with your manuscript** to the journal's Editorial Manager submission website.

☐ I have included a section, Conflict-of-Interest Statement, in the manuscript (applies even if there are no disclosures).

☒ I have no potential conflict of interest pertaining to this submission to *Cognitive Therapy and Research.*

Category for Disclosure	Description of Interest/Arrangement

Article Title Beyond Positive Affect: Discrete Positive Emotions Differentiate Social Anxiety Disorder from Major Depression

All Authors Angela A. Chin, Charles T. Taylor, Alison M. Sweet

Author Name Alison M Sweet

Author Signature [signature] Date 07/07/2022

Springer
http://www.springer.com/journal/10608

Cognitive Therapy and Research
Editor-in-Chief: Hofmann, S.G.
ISSN: 0147-5916 (print version)
ISSN: 1573-2819 (electronic version)
Journal no. 10608
