# Supplementary material for: Beyond Positive Affect: Discrete Positive Emotions Differentiate Major Depression from Social Anxiety Disorder
Source: Cognit Ther Res. 2023 Feb 13;47(3):377–85. doi: 10.1007/s10608-023-10355-y (PMC10164670; doi:10.1007/s10608-023-10355-y)

**Supplemental Materials**

**Supplemental Table 1**

*Between Groups Cohen’s d Effect Sizes*

|  | Group Comparisons | | | | | |
| --- | --- | --- | --- | --- | --- | --- |
| Discrete Positive Emotions | Control vs. SAD | Control vs. MDD | Control vs. Comorbid | SAD vs. MDD | SAD vs. Comorbid | MDD vs. Comorbid |
| Amusement | 0.99 | 1.50 | 1.59 | 0.45 | 0.54 | 0.09 |
| Awe | 1.02 | 1.64 | 1.55 | 0.54 | 0.46 | -0.09 |
| Gratitude | 1.00 | 1.41 | 1.36 | 0.27 | 0.37 | 0.13 |
| Hope | 1.74 | 2.39 | 2.64 | 0.45 | 0.62 | 0.18 |
| Inspiration | 1.53 | 2.42 | 2.24 | 0.69 | 0.55 | -0.15 |
| Interest | 1.35 | 2.49 | 2.16 | 0.77 | 0.60 | -0.15 |
| Joy | 1.62 | 2.77 | 3.06 | 0.76 | 0.96 | 0.23 |
| Love | 2.00 | 2.26 | 2.60 | 0.18 | 0.45 | 0.27 |
| Pride | 1.91 | 2.41 | 2.98 | 0.25 | 0.62 | 0.41 |
| Contentment | 1.63 | 2.43 | 2.45 | 0.51 | 0.62 | 0.17 |

*Note.* The effect sizes were calculated such that control vs. SAD reflects control minus SAD, control vs. MDD reflects control minus MDD, control vs. comorbid reflects control minus comorbid, etc. SAD = SAD only group. MDD = MDD only group. Comorbid = comorbid SAD and MDD group.

**Supplemental Table 2**

*Correlations Between Symptom Scores and Positive Emotion Ratings*

| Discrete Positive Emotions | LSAS | BDI | LSAS controlling for BDI | BDI controlling for LSAS |
| --- | --- | --- | --- | --- |
| Amusement | -.46** | -.56** | -.12 | -.39** |
| Awe | -.46** | -.50** | -.18* | -.29** |
| Gratitude | -.38** | -.47** | -.09 | -.31** |
| Hope | -.63** | -.67** | -.32** | -.42** |
| Inspiration | -.61** | -.65** | -.29** | -.41** |
| Interest | -.52** | -.67** | -.12 | -.50** |
| Joy | -.60** | -.73** | -.19* | -.55** |
| Love | -.63** | -.65** | -.33** | -.39** |
| Pride | -.68** | -.67** | -.41** | -.39** |
| Contentment | -.61** | -.68** | -.27** | -.45** |

*Note.* 223 participants of the 272 participants in the overall sample had both LSAS and BDI scores (*n* = 223).

** p < .01. ** p < .001.*

**Supplemental Figure 1**

*Frequency Distribution of LSAS Scores*


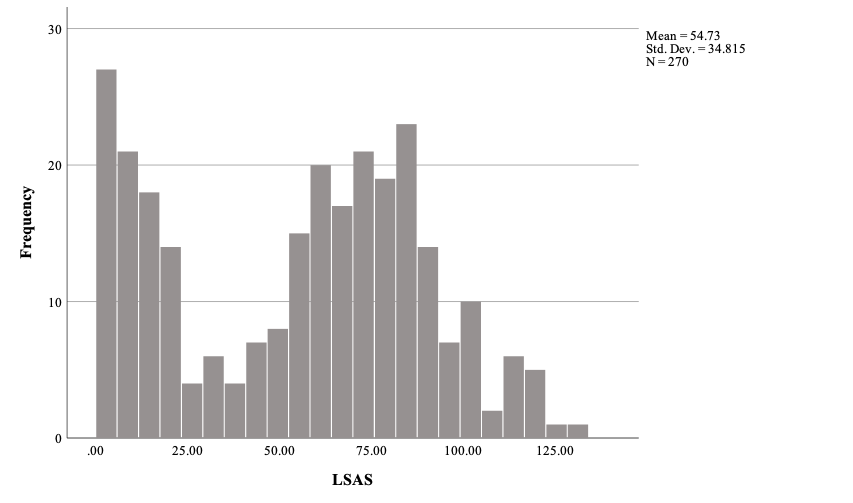


**Supplemental Figure 2**

*Frequency Distribution of BDI-II Scores*


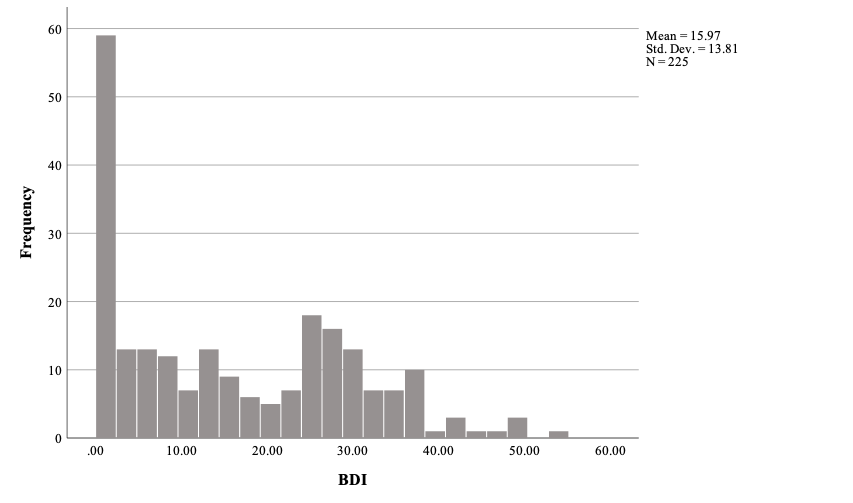

Supplement: Supplementary file 4 — Supplementary Material 4 [file 10608_2023_10355_MOESM4_ESM.docx]
